# Daily Experiences and Well-Being in Mid- and Later Life: Tests of Intra and Interindividual Moderators

**DOI:** 10.1093/geroni/igaf122.491

**Published:** 2025-12-31

**Authors:** Lydia Ong, Anna Kornadt, Shevaun Neupert

## Abstract

Stressful and positive experiences are common in daily life and associated with fluctuations in emotions, subjective cognition, and physiology, with implications for downstream health across mid- and later life. Daily diaries and ecological momentary assessment capture naturalistic experiences and provide insight into how these experiences covary with well-being within individuals. Importantly, these associations are shaped by how individuals interact with their environment. Thus, in this symposium, we present four studies examining intra and interindividual moderators of the links between daily experiences and well-being. First, Ong et al. examine the daily association between savoring and positive affect among adults in Switzerland (n = 108, Mage=73) and British Columbia (n = 178, Mage=47), testing whether individuals with more versus less positive views of aging differ in this relationship. In a sample of 1,125 US adults (Mage=62), Saruhanlioglu et al. investigate whether elevated positive affect buffers the relationship between daily stressors and memory lapses. Furthermore, Luchetti et al. look at the daily link between loneliness and subjective cognitive difficulties among 1,828 US adults (Mage=57), testing sociodemographic factors as moderators. Lastly, among 318 US parents (Mage=40), Pauly et al. consider whether personality moderates the associations of solitude with negative affect and diurnal cortisol slopes. Results from these studies demonstrate how daily experiences are linked with emotional, cognitive, and physiological well-being, and when or for whom associations might be buffered or strengthened. Findings will be discussed in the context of understanding how individual differences shape the impact of daily experiences on developmental trajectories across different timelines.

